# The regulation of cancer-associated thrombosis by podoplanin

**DOI:** 10.1016/j.tru.2024.100174

**Published:** 2024-04-17

**Authors:** Neha Gupta, Mohd Faiz Saifi, Kiesha Wilson, Yohei Hisada, Colin E. Evans

**Affiliations:** a Department of Biosciences, Faculty of Natural Sciences, Jamia Millia Islamia, New Delhi, India; b Department of Gastroenterology and Human Nutrition Unit, All India Institute of Medical Sciences, New Delhi, India; c Department of Pathology, Microbiology, and Immunology, University of South Carolina School of Medicine, Columbia, SC, USA; d Division of Hematology, Department of Medicine, University of North Carolina at Chapel Hill, Chapel Hill, NC, USA; e UNC Blood Research Center, University of North Carolina at Chapel Hill, Chapel Hill, NC, USA; f Department of Cell Biology and Anatomy, University of South Carolina School of Medicine, Columbia, SC, USA; g Cardiovascular Translational Research Center, University of South Carolina, Columbia, SC, USA; h Institute on Cardiovascular Disease Research, University of South Carolina, Columbia, USA; i Biomedical Engineering Program, College of Engineering and Computing, University of South Carolina, Columbia, SC, USA

**Keywords:** Cancer-associated thrombosis, Podoplanin, Thrombosis

## Abstract

The incidence of venous thromboembolism (VTE) in cancer patients is 4–9 fold higher compared with the general population. The mortality rate of patients with cancer and VTE is more than 2-fold greater versus cancer patients without VTE. Given that the pathophysiology of thrombosis in cancer is multi-faceted, investigations of the mechanisms that regulate cancer-associated thrombosis (CAT) could improve the understanding and treatment of CAT. These mechanisms include activation of the coagulation and fibrinolytic systems. Tumor cells activate coagulation by expressing procoagulant molecules, releasing pro-inflammatory and pro-angiogenic cytokines, and adhering to vascular and blood cells. Tumor-secreted and tissue factor-positive extracellular vesicles are another major driver of CAT, while emerging studies have discovered a role for podoplanin (PDPN) in intratumoral thrombosis, hyper-coagulation, and enhanced VTE risk. In this article, we will review studies of PDPN in CAT, which together suggest that PDPN contributes not only to cancer progression and metastasis, but also to CAT. PDPN may therefore represent an attractive putative target for therapies that aim to simultaneously reduce cancer progression and associated VTE.

## 1. Introduction

The association between cancer and thrombosis was first reported in 1823 by Jean-Baptiste Bouillaud, an association that was later recognized as Trousseau’s Syndrome by Armand Trousseau, who reported thrombophlebitis with an undiagnosed malignancy in 1865, tragically diagnosed the syndrome in himself, and died of gastric cancer [1]. Since then, many studies have investigated the incidence and pathobiology of cancer-associated thrombosis (CAT) [2,3]. CAT manifests as venous thromboembolism (VTE), or less commonly, arterial thromboembolism (ATE) [4–6]. The incidence of cancer-associated VTE vary widely according to study population, follow-up duration, and stage of cancer, but the overall incidence of VTE in cancer patients is 4–9 fold higher compared with the general population [7,8]. Some studies demonstrate that 20–30 % of all first VTE events are associated with cancer, and the mortality rate of patients with cancer and VTE is > 2-fold greater than those without VTE [3,8]. A recent age-stratified comparitive study demonstrated that in all age categories, the risk of VTE is higher in individuals with cancer than in those without [4]. A Danish population-based study estimated the cumulative incidence of VTE in 12 months from cancer diagnosis to be 3 % [9]. In addition, the authors reported that cancer patients have 9-fold higher risk of VTE than the general population. Strikingly, over the past two decades, the risk of VTE has increased by 3-fold overall and by 6-fold in those receiving chemotherapy or targeted therapies [9]. In fact, it has been found that chemotherapies cisplatin and topotecan increase the expression of genes that increase platelet aggregation [10]. Interestingly, the risk of VTE also varies according to primary tumor site, e.g., brain, lung, pancreatic, stomach, and ovarian cancer have a higher risk than breast and prostate cancer [8,9]. Gender, ethnicity, comorbidities, and treatment-associated factors also affect the risk of CAT [5,11–14].

The pathophysiology of thrombosis in cancer patients is complex and reflects multiple mechanisms related to the host response to malignancy. These mechanisms include activation of coagulation and fibrinolytic systems, and the acute inflammatory response [15–17]. For instance, tumor cells can activate coagulation by expressing procoagulant molecules; releasing pro-inflammatory and pro-angiogenic cytokines; and adhering to vascular and blood cells [18–21]. Tumor-secreted and tissue factor (TF)-positive extracellular vesicles are another major proponent of CAT, while emerging studies have discovered a role for podoplanin (PDPN) in intratumoral thrombosis, hyper-coagulation, and enhanced VTE risk [22–26]. In observational studies of various types of cancer, platelet aggregation is PDPN dependent [10,27–29]. While it is known that cancer-associated VTE is common, treatments for CAT are often associated with adverse side-effects including increased risk of bleeding. Currently, CAT patients are treated with anticoagulants, which prevent thrombus extension, but can also increase the risk of bleeding. Therefore, it is important to investigate specific mechanisms of CAT and search for novel therapeutic targets [30]. The current review aims to: (i) describe the structure and function of PDPN; (ii) summarize studies of the roles and mechanisms of PDPN in CAT; and (iii) identify directions for future research. To this end, we entered the following search terms into PubMed and Google Scholar: ‘cancer-associated thrombosis AND podoplanin’; ‘thrombosis AND podoplanin’; and ‘venous thromboembolism AND podoplanin’.

## 2. PDPN expression, structure, and binding

PDPN, also known as PA2.26, T1α, gp38, D2-40, E11 antigen, GP36, and aggrus, is a small transmembrane mucin-like glycoprotein whose sequence of amino acids is well conserved across all vertebrates [31]. PDPN is expressed in many cell types such as glomerular podocytes, osteocytes, type I alveolar cells, mesothelial cells, glia cells, neurons, lymphatic endothelial cells, and fibroblasts [31,32]. The expression of PDPN is upregulated in epithelial and mesenchymal cells during inflammation and cancer [33]. PDPN is concentrated in actin-rich microvilli and projections of plasma membranes such as lamellopodia and filopodia, where it colocalizes with ezrin, radixin, and moesin [34]. Interestingly, PDPN is also overexpressed in several kinds of tumors, including lung adenocarcinoma [35,36], small lung cell carcinoma [37], breast carcinoma [38–40], and perihilar cholangiocarcinoma [41].

**Fig. 1.**
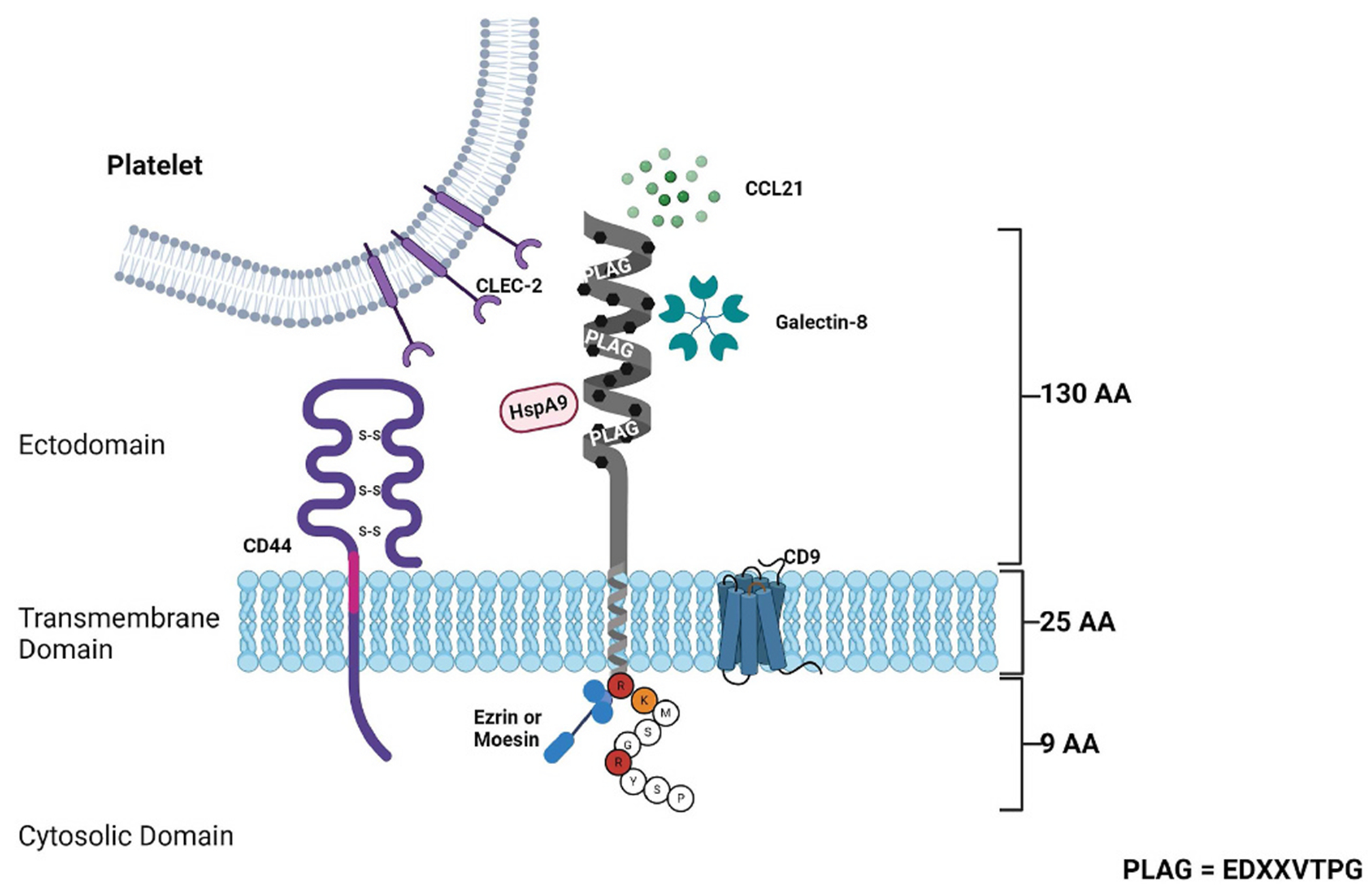
PDPN structure and binding. PDPN is a small transmembrane mucin-like glycoprotein made up of an extracellular domain (ectodomain), a transmembrane domain, and a short cytoplasmic tail. The PDPN ectodomain is a heavily *o*-glycosylated region made up of 130 amino acids. In this domain there are repeat sequences of EDXXVTPG, known as the PLAG domains. There are three represented PLAG regions from PLAG1 to PLAG3. C-type lectin-like receptors (CLEC-2) found on platelet cells can bind PDPN at the PLAG3 region and activate platelet aggregation. CCL21, a potent chemokine can also bind PDPN in the ectodomain and increase cell migration and adhesion; this is also true for Galectin-8, which binds in the ectodomain, and increases adhesion and haptotaxis of lymphatic endothelial cells. The last of the ectodomain ligands is heat shock protein AD (HspA9), which facilitates squamous cell carcinoma growth and invasiveness. The transmembrane domain of PDPN is made up of 25 amino acids and is the region where hyaluronan receptor CD44 can interact with PDPN, increasing epithelial cell motility. In an opposing manner, CD9 can interact with PDPN in the transmembrane region, leading to an inhibitory effect on platelet aggregation. The last portion of the PDPN structure is anchored in the cytosolic domain, is made up of 9 amino acids, and has the ability to bind Ezrin and Moesin, which both participate in cell invasion and movement.

Structurally, PDPN has a heavily glycosylated N-terminal extracellular domain (ectodomain) of approximately 130 amino acids followed by a hydrophobic single transmembrane domain of 25 amino acids, and a short intracellular cytoplasmic domain of 9 amino acids (Fig. 1). The ectodomain of PDPN contains sugar modifications at serine and threonine residues and contains galactose linked to β3 to N-acetyl-galactosamine (GalNAC), modified by the addition of sialic acid [42]. The ectodomain consists of a repeat sequence of EDXXVTPG, known as the PLAG1 to PLAG3 domains [42,43]. The transmembrane and intra-cellular cytoplasmic domains of PDPN are evolutionarily conserved (i.e., N -terminal motif GXXXG or G133IIVG137 in human), which are involved in helix-helix oligomerization [42]. As a substrate for presenilin-1/γ-secretase, the transmembrane domain of PDPN is cleaved (between V^150^ and V^151^ in humans) and the intracellular domain is released into the cytosol, although the functional relevance of this proteolysis remains to be investigated [44]. The cytoplasmic domain of PDPN comprises 9 amino acids (RKMS^157^GRYS^161^P) in humans [44], of which the first 2 residues (RK) are responsible for the binding of PDPN to ezrin and moesin [45]. The latter amino acid anchors the glycoprotein to the actin cytoskeleton [31,45].

PDPN primarily interacts with the C-type lectin-like receptor-2 (CLEC-2) and with the standard isoform of hyaluronase receptor CD44s at the surface of squamous cell carcinoma cells by alternative splicing [46]. The interaction between PDPN and CD44 is inhibited by glycosylation of the protein ectodomains of type-1 transmembrane glycoprotein [46,47]. The transmembrane and cytoplasmic domains of PDPN also participate in the interaction between CD44s and PDPN [48]. PDPN also binds with the tetraspanin cluster of differentiation 9 (CD9) and this interaction occurs through the transmembrane domains of tetraspanins at specialized membrane regions called tetraspanin enriched microdomains [49]. Several PDPN-binding ligands have been identified to date, which regulate various biological processes, including some that contribute directly to CAT. PDPN utilizes ligand binding activity to regulate tumor cell metastasis, invasion, and migration (Table 1).

## 3. PDPN function

### 3.1. PDPN and platelet aggregation

**Fig. 2.**
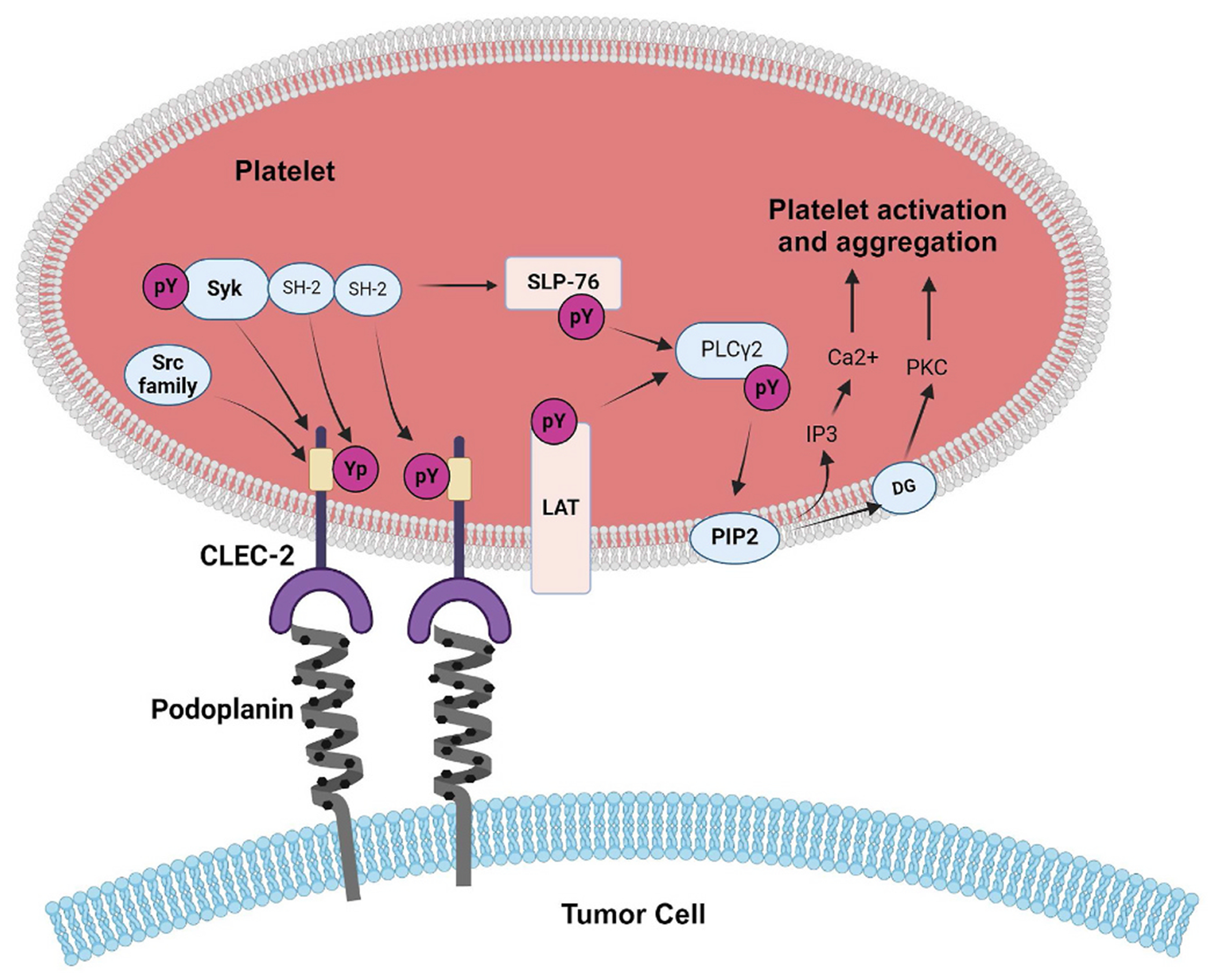
Regulation of platelet aggregation by PDPN. PDPN is upregulated on the surface of tumor cells and induces platelet activation and aggregation by interacting with the CLEC-2 receptor. PDPN interacts with CLEC-2 receptor via its PLAG domain. Upon ligation of PDPN to CLEC-2, the Src family kinases phosphorylate the solitary YxxL motif, hemi-ITAM. Subsequently, tyrosine kinase Syk increases its kinase activity by binding to the phosphorylated ITAM motifs through its SH2 domains. Syk phosphorylates the adaptor proteins LAT and SLP-76, which activates downstream signalling mediated by PLCy2. PLCy2 hydrolyses PIP2 to produce DG and IP3. These products in turn cause an increase in Ca2+ mobilization from the endoplasmic reticulum and the activation of PKC, which leads to platelet activation and aggregation. Abbreviations: PDPN, podoplanin; CLEC-2, C-type lectin like receptor-2; PLAG, platelet aggregation stimulating domain, hemi-ITAM, hemi-immunoreceptor tyrosine-based activation motif; SH2, Src homology 2; LAT, linker for activation of T cells; SLP-76, SH2 domain containing leucocyte protein of 76 kDa; PLCy2, phospholipase Cy2; PIP2, phosphatidylinositol 4,5 bisphosphate; DG, diacylglycerol; IP3, inositol 1,4,5 trisphosphate; PKC, protein kinase C.

PDPN induces platelet aggregation by interacting with CLEC-2 [67]. CLEC-2 has been identified in platelets as the receptor that is responsible for platelet aggregation when exposed to snake toxin, rhodocytin [67]. An anti-PDPN antibody, NZ-1, inhibits PDPN-induced platelet aggregation by binding to the PLAG domain of PDPN and interfering with the interaction between PDPN and CLEC-2 [68,69]. O-glycosylation at thr52 of the human PLAG-3 ectodomain of PDPN, or O-glycosylation at thr34 of the mouse PLAG-1 ectodomain of PDPN, is crucial for PDPN-dependent platelet aggregation [70,71]. In humans, PLAG4 of the ectodomain is distant from PLAG-1-3 and is involved in CLEC-2 interaction [70]. Crosslinking of CLEC-2 by rhodocytin or PDPN leads to tyrosine phosphorylation of a hemi-ITAM (YXXXL) (hemi-immunoreceptor tyrosine-based activation motif) of the CLEC-2 cytoplasmic tail by the Src kinases [72]. This promotes the binding of tyrosine kinase syk through its Src homology2 (SH2) phosphotyrosine binding domains, which is responsible for initiating a signaling cascade resulting in the phosphorylation of linker for activation of T cells (LAT) and SH2 domain-containing leucocyte protein (SLP)-76 adaptor proteins, and activation of effector enzymes including phospholipase CY2 (PLCy2), eventually resulting in platelet activation and aggregation [57,73]. Studies indicate that CLEC-2 recognizes both sialylated O-glycan and the adjacent peptide of PDPN [74]. Binding of PDPN to CLEC-2 is dependent upon sialic acid and O-glycans in the PLAG domain and plays a key role in platelet aggregation [43,56,67]. Mechanisms of PDPN-induced platelet aggregation are depicted in Fig. 2.

### 3.2. PDPN and metastasis

The inhibition of interaction between PDPN and CLEC-2 using an 8F11 monoclonal antibody for PDPN inhibits platelet aggregation and lung metastasis in mouse colon carcinoma [75]. PDPN-induced platelet aggregation is also involved in separation of the lymphatic and blood vasculatures during growth and development and in cancer metastasis [76]. PDPN is a key factor for platelet aggregation induced by the glioblastoma cell line, LN319 [77]. This was also found in treatment of glioblastoma cells, U87MG, with monoclonal anti-PDPN antibody, NZ-1, resulting in significant reduction of cell-platelet aggregation [78]. In experimental mouse models of hematogenous metastasis, PDPN promoted hematogenous metastasis to the lung and anti-PDPN antibody, NZ-1, suppressed the PDPN-CLEC-2 interaction and PDPN-induced pulmonary metastasis [74,79]. The binding of CD9 to tumor cells via PDPN reduces platelet aggregation and inhibits PDPN-induced metastasis in a mouse model of pulmonary metastasis [79]. The interaction between ERM proteins and PDPN is essential for PDPN-mediated rearrangement of the actin cytoskeleton, modulates small Rho GTPases, and facilitates epithelial-mesenchymal transition during embryogenesis and cancer progression [33,80]. Phosphorylation of serine residues in the cytosolic domain of PDPN also regulates its interaction with ERM proteins, thus modulating the anchoring of the glycoprotein to the cytoskeleton and activation of Rho GTPases [42]. In this case, two serine residues serve as the site of phosphorylation, i.e., S157 and S161 in human PDPN [54]. Phosphorylation of S157 and S161 suppresses PDPN-induced cell motility [46]. Recent studies have reported that cyclin-dependent kinase 5 (CDK5) and protein kinase A (PKA) can phosphorylate both serine residues intracellularly to decrease cell motility [81].

CLEC-2 is found in platelets and megakaryocytes in humans [82]. Some types of tumors also express the platelet activation receptor, CLEC-2 [83]. Hematogenous lung metastasis is facilitated by CLEC-2-PDPN interactions [83]. Reducing CLEC-2 levels suppressed hematogenous lung metastasis [79]. Moreover, this reduction in CLEC-2 improved the clinical outcomes of lung cancer patients, potentially by reducing thrombus formation in the lungs, and decreasing inflammation, anemia, and cachexia [56,73]. It is therefore possible that reducing thrombosis and inflammation through CLEC-2 depletion increases cancer patient survival.

Murine CLEC-2 is produced by platelets, megakaryocytes, natural killer cells, inflammatory macrophages, dendritic cells, peripheral neutrophils, and B cells [79], and this PDPN-binding ligand also regulates hemostasis and thrombosis [83,84]. CLEC-2 suppression prevented the spread of hematogenous tumors in a mouse model of hematogenous metastasis [79]. When B16F10 melanoma cells were co-cultured with wild-type platelets but not with platelets lacking CLEC-2, proliferation of the B16F10 cells increased. In CLEC-2-depleted animals, thrombus development in tumor vasculature was decreased and functional vascular density was increased. These findings suggest that a lack of CLEC-2 may prevent thrombus formation in tumor vessels and increase the density of functioning vessels, increasing oxygen and nutrients availability in tumors and indirectly promoting tumor growth.

## 4. Intratumoral regulation of PDPN

PDPN levels are increased in stromal cells of the tumor microenvironment, including lymphatic endothelial cells, immune cells [85], and cancer-associated fibroblasts (CAFs); these non-malignant cells have diverse roles in cancer progression (Table 2). In lung squamous cell carcinoma, CAFs expressing PDPN are linked with poor prognosis and tumor progression [35]. Co-injection of PDPN-positive vascular adventitial fibroblasts with lung adenocarcinoma cells results in increased tumor formation and metastasis [19]. PDPN-positive CAFs are also associated with an immunosuppressive tumor microenvironment, characterized by high levels of CD204+ tumor-associated macrophages and a low CD8/FOXP3 T-cell ratio [86]. Studies have also shown that FOXP3-positive regulatory T cells in the tumor microenvironment secrete immunosuppressive cytokines such as IL-10 and TGF-β, inhibiting cytotoxic T cells and enabling tumor cells to evade immune surveillance in various cancer types [87].

Within lymphatic endothelial cells, the transcription factor Prospero homeobox protein 1 (PROX-1) serves as the master regulator of PDPN transcription [89]. PDPN transcription can also be governed by other cytokines and transcription factors over the course of tumor progression. Hantusch et al. investigated PDPN transcription in 2 cell types: human osteoblast-like MG63 cells with high PDPN expression and Saos-2 cells with low PDPN expression [90]. Using an in vitro DNase I assay, they found several DNase I-protected regions within the PDPN promoter region spanning from bp 728 to 739, which were present in MG63 cells but absent in Saos-2 cells. Among these regions, they identified two specific Sp1/Sp3 binding sites that held the potential to regulate PDPN transcription. When Sp1 and Sp3 were overexpressed separately, they observed an increase in promoter activity and PDPN transcription in Saos-2 cells. Further confirmation through chromatin immunoprecipitation analysis established that Sp1/Sp3 were indeed recruited to the PDPN promoter. These findings suggest that Sp1/Sp3 members bind to their respective sites on the PDPN promoter, promoting PDPN transcription [90].

Durchdewald and colleagues conducted a study using a mouse model of skin carcinogenesis in K5–SOS–F transgenic mice to show that the Fos protein regulates PDPN [91]. They observed that PDPN expression in skin tumors was dependent on Fos, especially when induced by 12-*O*-tetradecanoylphorbol-13-acetate. When Fos was absent in mouse embryonic fibroblasts, the activity of the PDPN promoter decreased, but introducing Fos restored PDPN promoter activity. Additionally, their chromatin immunoprecipitation analysis confirmed that Fos directly binds to the 12-*O*-tetradecanoylphorbol-13-acetate-responsive element-like motif of the PDPN promoter. These findings highlighted the significance of the Fos-PDPN relationship in driving the transformation and progression of skin tumors [91].

In primary human glioblastoma and glioma cell lines, there is an inverse relationship between the levels of PDPN and phosphatase and tensin homolog (PTEN) [92]. In human glioma cells without PTEN, reintroducing wild-type PTEN, inhibiting PI3-kinase using LY294002, or blocking AP-1 activity with dominant-negative Jun and Fos all led to reductions in PDPN expression, indicating that the heightened expression of PDPN in human glioblastoma is linked to the loss of PTEN function and activation of PI3K-AKT-AP-1 signaling [92]. It has also been shown that low levels of oxygen in SJ-1 and U87 spheroids increase PDPN expression, suggesting that hypoxia may contribute to tumor progression and invasion through increases in PDPN [77]. There may also be a role for the epigenetic regulation of PDPN that deserves attention in future studies, for example through isocitrate dehydrogenase (IDH1) mutations in glioblastoma [93,94].

In oral squamous cell carcinoma, Mei and colleagues demonstrated that the ErbB3-binding protein-1 (EBP1) acts as a transcription factor that boosts PDPN transcription during tumor progression [95]. Their chromatin immunoprecipitation analysis unveiled EBP1 binding to the PDPN promoter near the Sp1/Sp3 site. Overexpression of EBP1 led to heightened PDPN transcription and invasiveness. Conversely, knocking down EBP1 suppressed PDPN transcription, invasiveness, and tumor formation in immunodeficient mice. Hence, EBP1 contributes to the increased expression of PDPN, playing a pivotal role in oral tumorigenesis [95]. PDPN expression can also be induced by interferon-γ (IFN-γ), transforming growth factor-β (TGF-β), and tumor necrosis factor-α (TNF-α) in squamous cell carcinoma cell lines [96]. Furthermore, when STAT1 (a key component in the IFN-γ pathway) was knocked down in squamous cell carcinoma cells, tumor cell invasion was suppressed in a subcutaneous tumor transplantation model [96]. The involvement of the TGF-βMAD pathway in PDPN expression has also been shown in oral/pharyngeal squamous cell carcinoma and fibrosarcoma cells. In these cases, TGF-β-induced PDPN expression was hindered by SMAD4 knockdown or treatment with a TGF-β type I receptor kinase inhibitor [97]. These findings underscore the significance of inflammatory cytokines produced by immune cells in stimulating PDPN expression, particularly at the invasive edge of tumors [98].

In gastric cancer tissues and cell lines, it was found that PDPN was upregulated and was associated with poor disease prognosis [99]. In this study, they found that PDPN positively regulated cell migration, invasion, and viability, and inhibited apoptosis, via the activation of ezrin [99]. Knockdown of PDPN resulted in the depression of ezrin in gastric cancer cell lines. Furthermore, PDPN-activated CAFs led to the increased expression of tumor-promoting cytokines IL-6, IL-8, and CCL2 [99]. While this study investigated PDPN expression changes in gastric cancer cells causing activation in CAFs, other studies have shown that PDPN expression in CAFs themselves, results in poor prognosis of patients with lung squamous cell carcinoma as well as gastric cancer progression [100,101]. For instance, it was demonstrated that PDPN-positive CAFs secreted periostin, which activates FAK/YAP signaling in gastric cancer cells, resulting in activation of the PI3K/AKT pathway, which positively regulates periostin. As a result, there is a positive feedback loop between PDPN-positive CAFs and cancer stem cells in gastric cancer, leading to gastric cancer progression [101].

## 5. PDPN and CAT

### 5.1. Clinical studies

Gi et al. conducted a retrospective study of VTE tissue from 180 VTE patient autopsies to investigate the histopathological features of CAT [102]. These VTE cases were collected between 1977 and 2019 and separated into patients with or without cancer. Major organs and tumors were analyzed for CAT. Immunohistochemical and immunofluorescence staining of VTE tissue showed that cancer cells (i.e., intra-thrombus cancer cell clusters or vascular wall invasions) were present in around 25 % of CAT cases. Almost 90 % of these cancer cells expressed TF and/or PDPN, along with necrosis, platelet aggregation, and fibrin formation. The expression of TF and PDPN in non-cancer cells and CD163-positive monocytes/macrophages was predominantly in the organizing thrombus area. The frequency of TF-positive monocytes/macrophages was higher in cancer-associated VTE tissue compared with cancer-free VTE. Co-expression of TF with CD163 was higher in thrombi from patients with CAT than in those without cancer, but the co-expression of PDPN with CD163 did not differ significantly between the two groups. A predominance of citrullinated histone H3 was observed in early organizing thrombi. The presence of intra-thrombus neutrophil extracellular traps was also associated with the early stages of thrombus organization, regardless of the presence or absence of cancer. Intriguingly, deep vein thrombosis in cancer patients showed greater organization than deep vein thrombosis in patients without cancer. These observations suggest that TF and PDPN in cancer cells and monocytes/macrophages induce CAT, and that cancer cells directly contribute to thrombogenesis in CAT [102].

A study by Wang et al. demonstrated higher incidence of VTE in non-small cell lung cancer patients with high PDPN expression than in those without PDPN expression [28]. In a 2-year follow-up period, the median time to 50 % mortality in PDPN-positive patients was 9.8 months, compared with 18.5 months in PDPN-negative patients. Higher PDPN expression in tumors of patients with pulmonary squamous cell carcinoma was also associated with an increased risk of VTE and poor prognosis [28]. These results signify that PDPN expression in tumor samples may help identify patients with a higher risk of VTE and mortality.

In a prospective cohort study, Riedl et al. explored the association of intratumoral PDPN expression with hypercoagulability, intratumoral platelet aggregation, VTE risk, and mortality in patients with primary brain tumors [27]. Immunohistochemical staining for PDPN and intratumoral platelet aggregates was performed in brain tumor specimens from 213 brain cancer patients enrolled in the Vienna Cancer and Thrombosis study with a follow-up period of 2 years. Intratumoral PDPN expression patterns were diffuse, heterogenous, and predominantly membrane-bound on tumor cells. There was no PDPN expression in normal cerebrum, cerebellum, or white matter. PDPN expression was positively correlated to the grade of intravascular platelet clusters. Expression of PDPN correlated with high D-dimer levels and low platelet count, along with high leukocyte counts, high prothrombin fragment 1 + 2, and high factor VIII activity. The risk of developing VTE was approximately 6 times higher in patients with high PDPN-expressing tumors compared with patients with PDPN-negative tumors. Cultured glioblastoma cells induced platelet aggregation via PDPN. During the 2-year observation period, patients with high PDPN-expressing tumors had a 2.6-fold higher risk of mortality compared with patients with PDPN-negative tumors after adjusting for age, sex, and tumor type. PDPN-positive primary glioblastoma cells induced aggregation of human platelets in vitro, which could be reversed with an anti-PDPN antibody. It was also shown that the risk of VTE in wildtype phenotypes with PDPN (6 month VTE risk of 18.2 %) was greater versus IDH1 mutant phenotypes without PDPN expression (6-month VTE risk of 0 %). Importantly, the expression of PDPN and IDH1 mutation in primary brain tumors appeared to be mutually exclusive. Similarly, another study by Birner et al. found an association between high PDPN expression and poor prognosis of cancer patients with brain tumors [103]. These studies together suggest that the risk of thrombosis is greater in cancer patients with PDPN-expressing tumors versus cancer patients with PDPN-negative tumors.

Patients with lung cancer are more at risk of thrombotic events compared with many other cancer types [104,105]. Zhang et al. showed significantly higher levels of D-dimer, fibrinogen, and platelets in the lung cancer cells of non-small cell lung carcinoma patients compared with healthy controls [106]. Additionally, soluble PDPN levels in plasma samples of non-small cell lung carcinoma patients were elevated in comparison to healthy individuals. This study provided indirect support for the notion that non-small cell lung carcinoma patients with increased soluble PDPN levels might be more susceptible to hypercoagulability and thrombosis, as evidenced by the strong positive associations between soluble PDPN and indices of coagulation.

It has been found that glioblastoma multiforme patients with VTE have lower platelet counts than controls and that PDPN tumor levels are associated with the probability of developing VTE [27]. Nadim and coworkers investigated the phenotypic landscapes of several glioblastoma multiforme cell populations to connect them with their coagulant phenotypes [107]. Based on single-cell RNA sequencing data, PDPN mRNA varied non-randomly across the individual cells that form human glioblastoma multiforme lesions. Notably, PDPN-high cell groups showed a larger expression profile for coagulant/inflammatory genes. In EGFRvIII (an oncogenic form of epidermal growth factor receptor)-driven glioma cells, the expression of PDPN was found to be under negative regulation by the chromatin modifier enhancer of zeste homolog 2 (EZH2). Notably, PDPN was detected in exosome-like extracellular vesicles released by glioma cells expressing PDPN. These extracellular vesicles are also found in varying amounts in the blood of patients with glioblastoma multiforme [108].

### 5.2. Experimental studies

To understand whether higher expression of tumor PDPN is associated with increased platelet activation and risk of VTE, Wang et al. investigated the effects of a human lung cancer cell line (NCI–H226) and a human melanoma cell line (C8161) - both of which express high levels of PDPN - on platelets in vitro and in vivo. In vitro, studies of these human malignant cell lines showed that targeted inhibition of PDPN by SZ168 (a monoclonal antibody) reduced platelet aggregation, and this treatment also decreased the incidence of VTE in mice [28]. Administration of SZ168-treated cancer cells gave rise to reduced tumorigenesis compared with mice receiving vehicle-treated cancer cells. Thrombi weight and platelet count were also reduced in mice receiving SZ168-treated cancer cells compared with vehicle controls. The authors went on to show that PDPN-high cancer cells induced PF4 secretion in platelet-rich plasma, which was inhibited by SZ168, and that platelet-specific P-selectin expression was inhibited by SZ168. These results indicate that PDPN expressed on tumor cells plays a significant role in platelet aggregation and activation in vitro, and that inhibition of tumor PDPN can suppress platelet activation and thrombotic incidence in mouse models of CAT. The study also showed that syk tyrosine kinase is phosphorylated when platelets interact with PDPN-high cancer cells in vitro. R406 (a syk inhibitor) inhibited platelet adhesion and aggregation in PDPN-high mouse melanoma cells in vitro, and partially inhibited venous thrombosis in tumor-bearing mice. In other words, PDPN-dependent platelet activation via syk tyrosine kinase signaling contributes to CAT, suggesting that PDPN offers a promising target for CAT.

To determine the levels of PDPN expression in oral squamous carcinoma cells, the cell lysates from 9 OSCC cell lines have been examined by Western blotting [109]. Five of the 9 cell lines expressed PDPN, i.e., OECM-1, OC2, TW2.6, HSC-3, and CAL27. Using an ectopic xenograft mouse model, this study showed that PDPN expression in OSCC tumors activates platelets, promotes intravascular platelet aggregation, and induces intratumoral platelet infiltration, all of which increase the coagulation state and reduce the survival of the mice with PDPN-expressing tumors. These findings together suggest that PDPN increases CAT and tumorigenesis, which could partially account for the aggressive phenotype of OSCC tumors with high PDPN expression [109].

Tumors that developed in mice after being injected with PDPN-expressing glioma cells triggered distinct profiles of circulating markers associated with thrombosis [107]. Furthermore, these effects were replicated by intravascular injections of GBM-derived extracellular vesicles. Authors of this study suggested that GBMs may consist of coagulant mosaics, comprising of both PDPN-expressing and non-expressing cell subpopulations, with coagulant phenotypes favoring the involvement of platelets in systemic CAT. The study also proposed that the detection and targeting of PDPN-expressing cells or their effects on platelets could mitigate GBM-associated systemic thrombosis [107].

Production of PDPN in the form of extracellular vesicles has been demonstrated in cases of ovarian cancer [10]. Ovarian cancer cells produce PDPN and release extracellular vesicles that are rich in PDPN [10]. The authors of the study reported that cisplatin and topotecan, two chemotherapy drugs frequently used to treat ovarian cancer, increase the expression of PDPN in cancer cells. PDPN expression in ovarian cancer cells promoted tumor formation, while suppression of PDPN expression resulted in smaller primary tumors in a mouse ovarian cancer model. In vitro, PDPN-negative cells (i.e., A2780 and OVCAR3) did not cause platelet aggregation, but PDPN-positive cells did (i.e., HeyA8, OVCAR4, and CAOV3) [10]. The findings of this study suggest that PDPN in ovarian cancer cells stimulates tumor growth and leads to venous thrombosis in mice. In a mouse model of Lewis lung carcinoma, Shirai et al. found that CAFs express PDPN and induce platelet aggregation in a manner that is dependent on CLEC-2 [110]. Tumor-bearing mice had increased levels of PDPN in plasma, and CLEC-2 depletion suppressed venous thrombosis in tumor-bearing but not tumor-free mice [110]. Authors of this study concluded that CAFs and extracellular vesicles from CAFs induce platelet aggregation and venous thrombosis via CLEC-2 [110].

## 6. Future perspectives

**Fig. 3.**
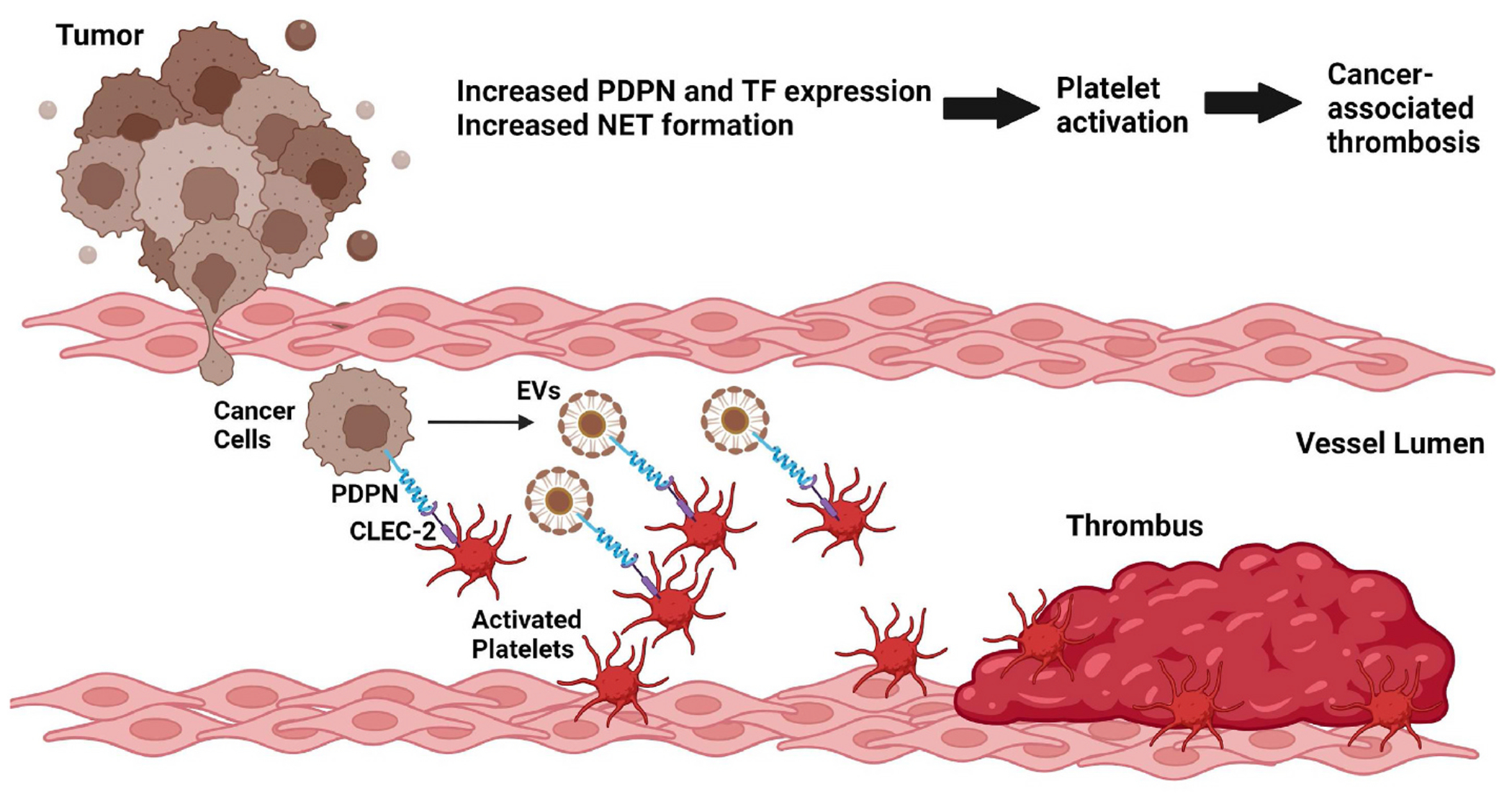
Mechanisms of CAT. Multiple factors expressed by cancer cells increase platelet aggregation, leading to thrombosis. Directly, tumors have high expression of TF and PDPN, which have significant roles in platelet activation and aggregation. PDPN expressed on the surface of cancer cells and their extracellular vesicles will bind to CLEC-2 on platelets, resulting in platelet and adhesion, which together with increases in other coagulant factors, leads to increased thrombosis.

Mechanisms of CAT include cancer cell-induced increases in TF expression, PDPN expression, neutrophil extracellular trap formation, and platelet activation (Fig. 3). Several studies have reported associations between PDPN levels and VTE in patients with brain and lung cancer. Future studies should investigate if there are associations between PDPN levels and VTE in patients with other types of cancer. PDPN is highly expressed in many types of cancer cells, particularly in brain cancer and squamous cell carcinoma. PDPN contributes to cancer progression and metastasis and is associated with reduced survival in cancer patients. In addition, PDPN-positive extracellular vesicles are released from PDPN-positive cancer cells into the circulation. These PDPN-positive cancer cells and extracellular vesicles aggregate platelets via CLEC-2 and may enhance thrombosis. Future studies should aim to elucidate the cell-specific mechanisms by which PDPN regulates cancer progression and associated thrombosis in different cancer types. It would also be useful to assess any potential side effects of targeting PDPN to prevent CAT.

## 7. Conclusions

PDPN contributes to not only VTE, but also tumor progression and metastasis. PDPN may be an attractive target to simultaneously reduce VTE and tumor progression in some types of cancer, perhaps without increasing the risk of bleeding.

## Figures and Tables

**Table 1:** PDPN domains, ligands and associated biological processes.

PDPN domain	Ligands	Biological functions	References
**Ectodomain**	C-type lectin-like receptor 2	Wound repair	[50]
		Lymphangiogenesis	[51]
		Development of lymphatic vasculature	[52]
		Thrombosis	[53]
		Vascular development and homeostasis	[54]
		Cancer invasion and metastasis	[55]
		Platelet activation	[56]
		Platelet biogenesis	[57]
	C–C motif chemokine ligand 21	Development of natural Treg cells	[58]
		Immune response	[59]
		Tumor immune escape	[60]
	Heat-shock protein A9	Cell growth and invasiveness	[61]
	Galectin-8	Lymphangiogenesis	[62]
**Transmembrane domain**	Cluster of differentiation 9	Inhibition of PDPN-CLEC-2 interaction	[49]
	Cluster of differentiation 44	Directional cell migration	[46]
			
**Cytosolic domain**	Ezrin/radixin/moesin	Endothelial to mesenchymal transition	[33]
		Heart development	[63]
		Cancer cell invasion	[64]
		Immune response	[32]
		Cancer cell invasion and metastasis	[65]
	Matrix metalloproteinase 14	Cancer cell invasion and metastasis	[66]

**Table 2:** Roles of PDPN-expressing CAFs in tumor progression.

Cancer Type	Function	References
Lung adenocarcinoma	Promotes tumorigenicity and chemoresistance	[35,36]
Perihilar cholangiocarcinoma	Promotes tumor cell migration	[40]
Small cell lung carcinoma	Inhibits the development of tumor cells	[37]
Breast carcinoma	Promotes immunosuppression	[38,39]
Pancreatic ductal carcinoma	Promotes tumor cell invasiveness	[88]
